# Bing‐Neel syndrome

**DOI:** 10.1002/jha2.585

**Published:** 2022-10-02

**Authors:** Katelynn Davis, William H. Sharfman, Ivo M. B. Francischetti

**Affiliations:** ^1^ Department of Pathology Johns Hopkins University School of Medicine Baltimore Maryland USA; ^2^ The Sidney Kimmel Comprehensive Cancer Center at Johns Hopkins Baltimore Maryland USA

**Keywords:** ibrutinib, lymphoplasmacytic lymphoma, Waldenström's macroglobulinemia

## 1 BING‐NEEL SYNDROME

**FIGURE 1:**
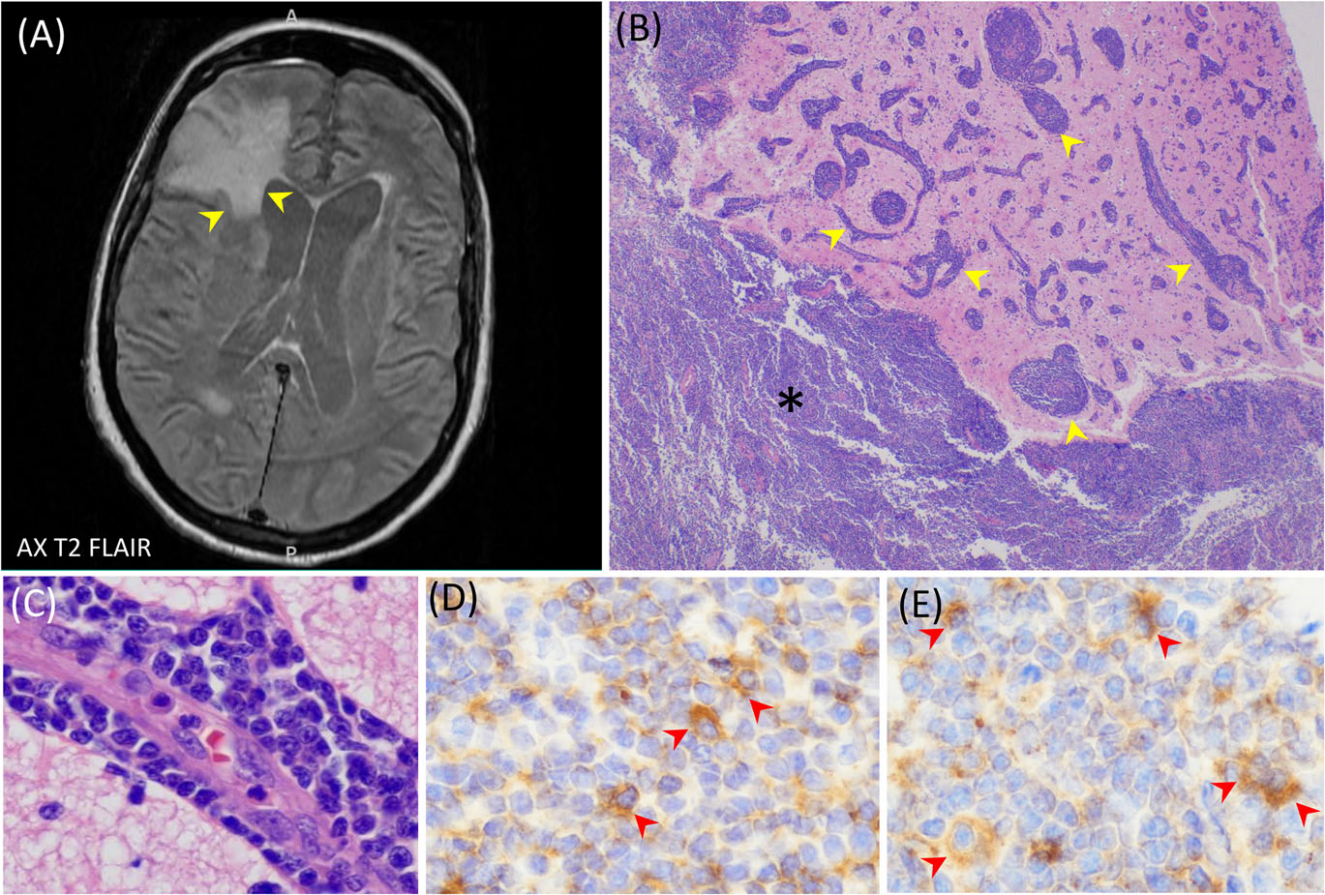
(A) A brain magnetic resonance imaging (MRI) shows a right frontal lobe cortical‐based enhancing mass (yellow arrowheads) with associated dural enhancement. (B) Stereotactic biopsy. Large area showing a diffuse (asterisk) and perivascular proliferation (yellow arrowheads) by small lymphocytes with round nuclei and clumped chromatin and admixed plasma(cytoid) cells (x20). (C) Dense perivascular infiltrates (x400). Immunohistochemistry shows plasma(cytoid) cells positive for (D) immunoglobulin M (IgM) (red arrowheads) and (E) kappa‐light chain (red arrowheads)

A 78‐year‐old man presented with a medical history of immunoglobulin M (IgM) kappa monoclonal gammopathy first detected by serum protein electrophoresis (SPEP) over two decades ago. He was diagnosed with lymphoplasmacytic lymphoma (LPL) with minimal marrow involvement, consistent with Waldenström macroglobulinemia (WM). WM is typically associated with MYD88 L265P somatic mutation. He was treated with chemotherapy and rituximab followed by multiple cycles of bortezomib and an extended course of ibrutinib. About three years ago, he developed axillary, retroperitoneal, pelvic, and inguinal lymphadenopathy consistent with disease progression, despite therapy. Over the last few months, his spouse noticed his gradual mental deterioration including increased confusion, difficulty walking, lethargy, and incontinence. SPEP showed a spike in the gamma region and serum immunofixation (IEF) identified IgM kappa monoclonal gammopathy. Serum IgM was 694 mg/dl. A brain magnetic resonance imaging showed a 2.8 cm right frontal lobe cortical‐based enhancing mass (T2/FLAIR hyperintense signal) with associated dural enhancement (Figure 1A). Brain and dural biopsies were performed and revealed the involvement by low‐grade B‐cell lymphoma with plasmacytic differentiation in diffuse (Figure 1B, asterisk) and perivascular distributions (Figure 1B, arrowheads; Figure 1C). Immunostains revealed that the B‐cell proliferation was positive for CD20 (not shown), with a subset of plasmacytoid cells expressing IgM (Figure 1D), and Kappa‐light chain (Figure 1E). Central nervous system (CNS) involvement by LPL, known as Bing‐Neel syndrome, is a rare manifestation of WM that usually presents as a feature of relapsing disease involving the CNS.

## CONFLICT OF INTEREST

The authors declare they have no conflicts of interest.

## ETHICS STATEMENT

No research on humans was performed in this study.

